# Epigenome-wide methylation and progression to high-grade cervical intraepithelial neoplasia (CIN2+): a prospective cohort study in the United States

**DOI:** 10.1186/s12885-023-11518-6

**Published:** 2023-11-06

**Authors:** Alexandra Bukowski, Cathrine Hoyo, Nadja A. Vielot, Misa Graff, Michael R. Kosorok, Wendy R. Brewster, Rachel L. Maguire, Susan K. Murphy, Belinda Nedjai, Efthymios Ladoukakis, Kari E. North, Jennifer S. Smith

**Affiliations:** 1 Department of Epidemiology, University of North Carolina at Chapel Hill, 60 Bondurant Hall, Chapel Hill, NC 27599 USA; 2 Department of Biological Sciences, Center for Human Health and the Environment, North Carolina State University, Raleigh, NC 27695 USA; 3 Department of Family Medicine, University of North Carolina at Chapel Hill, Chapel Hill, NC 27599 USA; 4 Department of Biostatistics, University of North Carolina at Chapel Hill, Chapel Hill, NC 27599 USA; 5 Department of Obstetrics and Gynecology, University of North Carolina at Chapel Hill, Chapel Hill, NC 27599 USA; 6 Department of Obstetrics and Gynecology, Duke University Medical Center, Durham, NC 27701 USA; 7 Centre for Prevention, Detection and Diagnosis, Wolfson Institute of Population Health, Queen Mary University London, London, UK; 8 University of North Carolina Lineberger Comprehensive Cancer Center, Chapel Hill, NC 27599 USA

**Keywords:** Cervical cancer, Cancer surveillance and screening, DNA methylation, Epigenomics, Epigenetics, Cancer risk, Pre-neoplasia

## Abstract

**Background:**

Methylation levels may be associated with and serve as markers to predict risk of progression of precancerous cervical lesions. We conducted an epigenome-wide association study (EWAS) of CpG methylation and progression to high-grade cervical intraepithelial neoplasia (CIN2 +) following an abnormal screening test.

**Methods:**

A prospective US cohort of 289 colposcopy patients with normal or CIN1 enrollment histology was assessed. Baseline cervical sample DNA was analyzed using Illumina HumanMethylation 450K (*n* = 76) or EPIC 850K (*n* = 213) arrays. Participants returned at provider-recommended intervals and were followed up to 5 years via medical records. We assessed continuous CpG M values for 9 cervical cancer-associated genes and time-to-progression to CIN2+. We estimated CpG-specific time-to-event ratios (TTER) and hazard ratios using adjusted, interval-censored Weibull accelerated failure time models. We also conducted an exploratory EWAS to identify novel CpGs with false discovery rate (FDR) < 0.05.

**Results:**

At enrollment, median age was 29.2 years; 64.0% were high-risk HPV-positive, and 54.3% were non-white. During follow-up (median 24.4 months), 15 participants progressed to CIN2+. Greater methylation levels were associated with a shorter time-to-CIN2+ for *CADM1* cg03505501 (TTER = 0.28; 95%CI 0.12, 0.63; FDR = 0.03) and *RARB* Cluster 1 (TTER = 0.46; 95% CI 0.29, 0.71; FDR = 0.01). There was evidence of similar trends for *DAPK1* cg14286732, *PAX1* cg07213060, and *PAX1* Cluster 1. The EWAS detected 336 novel progression-associated CpGs, including those located in CpG islands associated with genes *FGF22, TOX, COL18A1, GPM6A, XAB2, TIMP2, GSPT1, NR4A2*, and *APBB1IP*.

**Conclusions:**

Using prospective time-to-event data, we detected associations between *CADM1*-, *DAPK1*-, *PAX1*-, and *RARB*-related CpGs and cervical disease progression, and we identified novel progression-associated CpGs.

**Impact:**

Methylation levels at novel CpG sites may help identify individuals with ≤CIN1 histology at higher risk of progression to CIN2+ and inform risk-based cervical cancer screening guidelines.

**Supplementary Information:**

The online version contains supplementary material available at 10.1186/s12885-023-11518-6.

## Introduction

DNA methylation patterns throughout the human genome may serve as clinical biomarkers to predict the progression of cervical precancerous lesions to cancerous lesions, thereby improving screening algorithms. In the United States (US), the addition of high-risk human papillomavirus (hrHPV) co-testing to traditional Papanicolaou (Pap) cytology testing has improved risk stratification of cervical abnormalities [1, 2]. However, millions of abnormal results require multiple rounds of follow-up testing to monitor disease progression [2–4]. In particular, individuals with low-grade cervical intraepithelial neoplasia or less severe (≤CIN1) return for surveillance at regular intervals until the resolution of their abnormality; if their cervical lesion progresses to high-grade cervical intraepithelial neoplasia (CIN2/CIN3), treatment is recommended to prevent further progression. This lengthy surveillance period poses a high financial and logistical burden to both patients and healthcare systems, may subject individuals to over-testing and over-treatment, and increases loss to follow-up, especially among those with poor access to care [4–8]. Reducing follow-up visits and biopsies needed following an abnormal screening test—particularly for those classified as low-grade—can improve the efficiency of screening programs. Methylation at 5’-cytosine-phosphate-guanine-3’ (CpG) sites in cervical samples are promising biomarkers to improve risk stratification—and therefore clinical decision-making algorithms—for low-grade screening-detected cervical abnormalities.

Few studies have prospectively assessed methylation-associated progression risk of ≤CIN1 in screening populations. Many cross-sectional and case–control studies have been performed to assess methylation patterns in cervical cancer samples versus normal controls [9–15], but there have been far fewer prospective studies of methylation-associated progression risk, which could improve risk stratification methods used to inform cervical cancer screening guidelines. Methylation markers have also been studied more extensively in high-grade or cancerous cervical lesions, while investigation of low-grade precancerous lesions has lagged farther behind. This is despite the fact that low-grade lesions are the most common abnormalities detected, and their surveillance comprises the vast majority of cervical cancer screening activities. Additionally, initial methylation studies have targeted smaller numbers of methylation sites for assessment, and few have performed epigenome-scale analyses of lower-grade or early precancerous lesions. Finally, of the studies that have prospectively assessed epigenome-scale methylation patterns, very few have been performed in multiracial US populations. It is important to ensure diverse study populations—both geographically and demographically—in methylation biomarker studies, as both cervical disease risk and methylation profiles can vary by location and sociodemographic characteristics [16–18]. Inclusion of black, indigenous, and people of color (BIPOC) in screening studies will also optimally inform risk-based clinical decision-making, since these groups bear a disproportionate burden of cervical cancer morbidity and mortality [19, 20] and their exclusion can contribute to health disparities [21, 22]. Thus, there is a need to perform prospective, epigenome-wide analyses in low-grade screening-detected cervical lesions in diverse US populations in order to most appropriately inform risk-based national screening algorithms.

The purpose of this study was to investigate methylation biomarkers in samples collected during routine cervical cancer screening. Specifically, we assessed associations between methylation levels in liquid-based cervical cytology samples and the future risk of progression to CIN2+ during follow-up for screening-detected cervical abnormalities. Our analysis was comprised of individuals with ≤CIN1 from the Cervical Intraepithelial Neoplasia Cohort Study (CINCS), a US-based prospective, multi-racial cohort of women presenting to colposcopy following abnormal cervical cancer screening results. Our primary objective was to assess associations between methylation levels at a set of pre-selected CpG sites related to genes that have previously been associated with the development of cervical cancer and the prospective risk of developing CIN2+ over five years. Our secondary objective was an exploratory analysis of these associations at the methylome level—for all CpG sites in an epigenome-wide methylation array.

## Materials and methods

### Study population

This is an analysis of secondary data from the Cervical Intraepithelial Neoplasia Cohort Study (CINCS). CINCS is a prospective clinical cohort of 1,372 women with abnormal cervical cancer screening results who presented for follow-up referral colposcopy in Durham, North Carolina, between September 2010 and March 2016**.** All those presenting for colposcopy had a previously abnormal cervical cancer screening test—by cytology or cytology/HPV co-testing—that triggered referral to colposcopy in accordance with U.S. national guidelines [23]. Colposcopy clinic attendees at 10 Duke University clinics were invited to participate, as previously described [24]. Participants were study-eligible if they were 21–79 years old, English or Spanish speakers, new visitors to the clinic, and provided written consent. Patients were excluded if they had previous treatment for cervical lesions [i.e., cold knife conization (CKC), loop electrosurgical excision procedure (LEEP), cryotherapy], had a hysterectomy, had moved out of the study area, or did not intend to receive follow-up care at a participating clinic.

At enrollment, all participants underwent a physician-directed pelvic exam, which included collection of exfoliated cervical cells (for cytology, HPV DNA genotyping, and DNA methylation array testing) and a colposcopy examination with biopsies (for histology). An endocervical component (ECC) was collected on anyone with insufficient transformation zone due to anatomic variability from person to person, or a Pap cytology result of atypical glandular cells (AGC), adenocarcinoma in situ (AIS), or high-grade squamous intraepithelial lesion (HSIL). Cervical cytology was also collected at all follow-up visits approximately annually for up to 5 years. Colposcopy-directed biopsies and ECC were only collected at follow-up visits if abnormalities were visualized during the colposcopy exam, per conservative clinical practice. All colposcopies were performed by experienced colposcopists affiliated with Duke University. Abnormal cytology and histology results during follow-up were managed per U.S. national clinical guidelines [23]. Study staff administered study questionnaires to participants at enrollment and each follow-up visit to collect socio-demographic, behavioral, and clinical characteristics.

This study was conducted in accordance with ethical guidelines and approval was granted by the Institutional Review Boards at Duke University (Durham, NC, US; IRB Pro00022943), North Carolina State University (Raleigh, NC, US; IRB 3565) and University of North Carolina (Chapel Hill, NC, US; IRB 15–2364 and 321403).

### Ascertainment of cervical cytology, histology, and HPV typing

Cervical cytology was ascertained from exfoliated cervical specimens collected at each study visit via ThinPrep® liquid-based cytology (LBC) (Hologic Corporation, Marlborough, MA, US). Cervical exfoliated specimens were suspended in a ThinPrep® vial containing proprietary fluid with at least 50% methanol (Cytyc®, Marlborough, MA, US). Cytology was evaluated by the Duke University Hospital Anatomic Pathology Laboratory according to Bethesda criteria [25]. Residual specimens were stored at 4 °C prior to HPV DNA and methylation testing.

Cervical histology was ascertained from colposcopy-directed biopsy specimens at enrollment for all participants and at follow-up per clinical indication. Biopsy results were reviewed and graded for severity by Duke-affiliated pathologists, and specimens were tested for adequacy per 2012 ASCCP guidelines [23]. Cytology and histology information were abstracted from patient medical records.

HPV DNA was detected from exfoliated cervical cells collected at enrollment [24]. Following DNA extraction, PGMY09/PGMY11 primers were used in PCR to target a 450-bp region of the HPV L1 genome. Amplification of the human β-globin gene was included as an internal control to ensure sample sufficiency. HPV-positive specimens were subsequently genotyped using the HPV Linear Array® (Roche Diagnostics, Branchburg, NJ, US). This assay detects 13 hrHPV types (16,18, 31, 33, 35, 39, 45, 51, 52, 56, 58, 59, and 68) and 24 low-risk HPV (lrHPV) types (6, 11, 26, 40, 42, 53, 54, 61, 62, 64, 66, 67, 69, 70, 71, 72, 73, 81, 82, 83, 84, Is39, and cp6108).

### Exposure assessment: DNA methylation

DNA was extracted from LBC cell pellets obtained from the exfoliated cervical samples collected at enrollment. DNA methylation was analyzed in three batches. The first batch (*N* = 98 tested) underwent methylation testing with the Illumina Infinium HumanMethylation450 BeadChip array in 2017. The second (*N* = 100 tested) and third (*N* = 114 plus nine technical replicates tested) batches underwent testing with the Illumina Infinium MethylationEPIC BeadChip array in early and mid-2022, respectively [26]. All three batches underwent quality control (QC), data processing, and statistical analyses separately; subsequently, the three sets of results were combined via meta-analysis. Illumina methylation array data quality control and processing steps are detailed in the Supplementary document, along with a schematic summary of the processing pipeline (Supplementary Figure S1) [27].

#### Targeted gene analyses

For “targeted” analyses, we considered 10 pre-selected genes for their known associations with cervical pre-cancer progression or severity in a recent meta-analysis: *CADM1*, *CCNA1*, *CDH1*, *CDKN2A*, *DAPK1*, *FHIT*, *MAL*, *PAX1*, *RARB*, and *RASSF1 *[15]. All CpG sites associated with each gene were identified using the Illumina annotation datasets. Of note, no CpG sites remained in the dataset that were associated with *CDKN2A*, thus only nine genes were included in the following analytic steps. CpG correlation clusters were created for each of the nine genes of interest. Pairwise Pearson correlations between all CpGs for an individual gene were estimated, and CpGs were clustered together if they exhibited > 0.5 correlation with other CpGs associated with the gene; otherwise, the CpG site was analyzed individually. Multiple clusters within one gene were possible; this occurred in some cases where a subset of CpG sites were positively correlated with each other but negatively correlated with other CpG sites within the same gene. CpG clusters were created for each participant by computing the median M value of the component CpG sites in the cluster. Continuous M values for each site or cluster were used in statistical models.

#### Epigenome-wide association study (EWAS)

All CpGs shared among all three batches were included in EWAS analyses. These CpG sites were not clustered prior to analyses. Continuous M values were used in statistical models.

### Outcome assessment: Incident CIN2+ 

Methods for outcome ascertainment have been previously described [28]. Briefly, the outcome of interest was a diagnosis of CIN2+ (“progression”) at any point during the follow-up period. Incident CIN2+ was defined as a histologic diagnosis of CIN2+ (CIN2, CIN2-3, CIN3, or invasive cervical cancer). Outcome status was determined on the earliest date of the progression event, and participants were right-censored from further follow-up thereafter. For participants who received treatment during follow-up (LEEP, CKC, cryotherapy, or hysterectomy), the more severe histologic result between the colposcopy-directed biopsy and the excisional treatment specimen was used for the final follow-up diagnosis. Those receiving treatment during follow-up were right-censored from further follow-up on the date of treatment.

Time-to-progression was measured in person-months from the date of study enrollment to the date of progression. Participants contributed person-months up to the time of progression, to the date of treatment, or to the date of their last attended clinical study visit, whichever occurred first. Progression events were considered interval-censored events, since we knew they occurred at some point in the interval between the previous visit and the visit at which progression was detected. Thus, for participants who progressed during follow-up, their progression interval was defined on the left as “the time from enrollment to their last clinical visit where they hadn’t progressed” and on the right as “the time from enrollment to the clinical visit where they were found to have progressed”.

### Covariate assessment

Age at enrollment was calculated as time between date of birth in the medical chart and date of study enrollment. Race was ascertained from participant questionnaires collected at the enrollment clinic visit. Participants self-classified their race as “Black or African American”, “Non-Hispanic White”, “Hispanic White”, “Asian/Pacific Islander”, “American Indian/Native American”, Biracial or Multiracial”, or “Other”; they had the option to specify their racial identity in an open-ended question. Two surrogate variables were created to capture latent variation in the data with respect to outcome status (progression to CIN2+ vs. no progression) using the *sva* package in R. These two surrogate variables are meant to capture variation due to unknown or unmeasured sources of biological heterogeneity in the data.

### Analytic sample

**Fig. 1:**
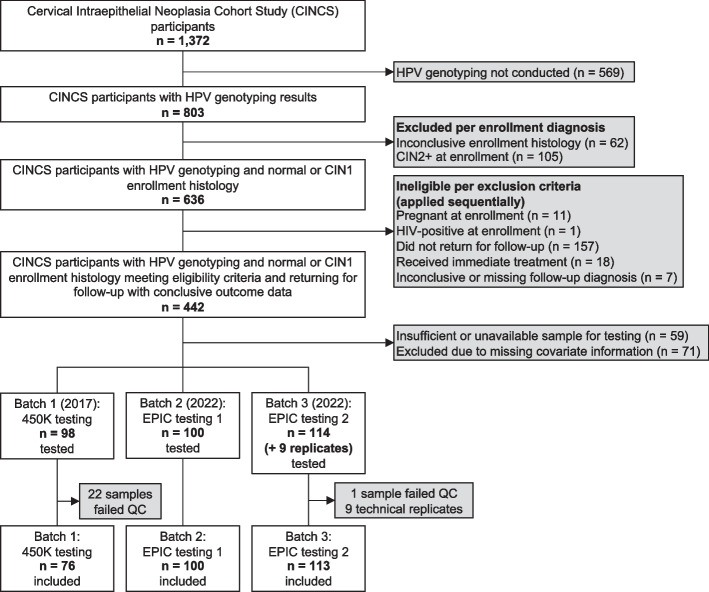
Flowchart for 289 CINCS participants included in analytic sample. Eligibility and inclusion criteria for a secondary data analysis of the Cervical Intraepithelial Neoplasia Cohort Study (CINCS), based in Durham, North Carolina, US

This analysis included CINCS participants with normal or CIN1 histology at study enrollment who had HPV genotyping and DNA methylation array data, were not pregnant or HIV-positive at enrollment, reported no history of HPV vaccination, and returned for at least one follow-up visit (Fig. 1). Of 1,372 enrolled CINCS participants, 803 had HPV DNA laboratory results; these 803 constitute the parent study sample available for further testing and analyses. Of these, 62 women had inconclusive enrollment histology and were excluded. An additional 105 with CIN2+ at enrollment were excluded. Of the remaining 636 participants, 11 participants who were pregnant, 1 who was HIV-positive, 157 who did not return for a follow-up visit, 18 who received immediate treatment, and 7 with an inconclusive or missing follow-up diagnosis were excluded. Of the remaining 442 participants, 59 had insufficient or unavailable sample for testing and 71 were excluded from testing due to incomplete covariate information necessary for processing the methylation data. This left 312 participants whose samples underwent methylation array testing for this analysis. Of these, 23 samples failed quality control metrics, leaving a final analytic sample of 289 participants.

### Statistical analysis

Descriptive statistics summarized the baseline distribution of socio-demographic characteristics and histologic/cytologic outcomes. Pearson’s chi-square test was used to compare a.) characteristics stratified by enrollment histology (no CIN vs. CIN1), and b.) characteristics of those retained in the study versus those who were lost to follow-up to assess potential bias due to attrition.

#### Targeted analysis

For the targeted gene-based analysis, Weibull-distributed accelerated failure time (AFT) models were used to model the association between the continuous methylation M value at each individual CpG cluster/site and time-to-progression to CIN2+ using interval-censored data [29–31]. The Weibull AFT model was chosen due to the small outcome numbers in our sample, the relative flexibility of the Weibull model over an exponential model, and the ability to re-parameterize the Weibull model to estimate hazard ratios (HRs).

All AFT models were adjusted for age, race, and two surrogate variables to control for latent sources of variation in the data. Race was included as a two-category variable (“non-Hispanic White race” vs. “race other than non-Hispanic White”); racial categories were collapsed due to small numbers in categories other than “non-Hispanic White” and “Black or African American”. Though collapsing causes loss of information, including this two-category race variable still improved genomic inflation in model output. Two categorizations for smoking status—current vs. former smoker or ever vs. never smoker—were also considered for inclusion in statistical models, but outcome numbers were too small after additionally stratifying by race, so neither was included in final models. We did not stratify by HPV type due to small outcome numbers.

Targeted analyses were performed in each of the three batches separately, and the three sets of results were meta-analyzed using EasyStrata in R [32]. EasyStrata conducts an inverse-variance weighted meta-analysis of input strata and returns pooled overall effects, standard errors (SEs), and *p*-values [32, 33]. False discovery rates (FDRs) were estimated from the meta-analyzed *p*-values using the Benjamini–Hochberg method.

The meta-analysis AFT model parameter corresponding to methylation was exponentiated to estimate a time-to-event ratio (TTER); the TTER corresponds to the multiplicative change in time-to-progression with each one-unit increase in continuous methylation M value. The model parameter corresponding to the methylation value was also converted to its corresponding HR by reparametrizing the AFT model in terms of a hazard function. The standard errors for the HR were estimated from the AFT model output using the delta method and were used to construct the 95% confidence interval (CI). AFT models were fit using the *survival* package in R [34], and the model output was converted to proportional hazards parameters using the *ConvertWeibull()* function in the *SurvRegCensCov* package [35]. For targeted CpG sites with methylation parameter *p*-values < 0.1, risk curves representing the cumulative probability of progression to CIN2+ over time were constructed using AFT model output for 10^th^, 50^th^, and 90^th^ percentiles of methylation M values. This cumulative probability of progression represents the absolute risk of CIN2+ estimated from these parametric AFT models.

#### Exploratory EWAS

For the EWAS analysis, similar Weibull-distributed AFT models (adjusted for age, race, and two surrogate variables) were fit for each CpG site in the methylation array. These models were fit for all CpGs in each of the three batches separately and were meta-analyzed using EasyStrata. Meta-analysis *p*-values were adjusted to account for multiple comparisons via the Benjamini & Hochberg (BH) method. We assessed genomic inflation using Q-Q plots of *p*-values from the EWAS output and lambda values comparing expected vs. observed *p*-values (with the goal of getting lambda close to 1). For all CpG sites with an epigenome-wide BH-adjusted *p*-value < 0.05 (false discovery rate, or FDR, < 0.05), we mapped the CpGs to their corresponding genes and chromosomal locations using Illumina annotation files. Finally, a gene set enrichment analysis (GSEA) was conducted using the *missMethyl* R package to test for Gene Ontology (GO) terms and Kyoto Encyclopedia of Genes and Genomes (KEGG) pathway enrichment in our results [36].

#### Methylation risk scores

We proposed a methylation risk score (MRS) using a pooled dataset of all three batches of methylation data, using an EWAS approach. We constructed the MRS using a weighted sum approach with internal weights [37]. To do this, we randomly split the pooled data set 50–50 into a training set (*N* = 154) and a test set (*N* = 135) using the *sample()* function in base R. A 50–50 split was chosen due to the small number of outcomes; a more uneven split may have led to model instability in the smaller set. The training set was used to estimate the internal weights, which were then used in the test set to calculate the weighted MRS. In the training set, we fit AFT models for each CpG site, adjusted for age, race, two surrogate variables, and a three-level “batch” variable. We created two MRS versions: All CpG sites with Bonferroni-adjusted *p* < 0.05 in the training set were included in the first MRS (for a more conservative selection criterion) and those sites with FDR < 0.05 were included in the second MRS. The regression beta parameter value for each CpG from the training set served as the “weight” in the MRS. Then, for each participant $i$ in the test set, the MRS was constructed as a weighted sum of M values of all $k$ detected CpG sites: ${MRS}_{i}={weight}_{{CpG}_{1}}*{M}_{{CpG}_{1i}}+\dots +{weight}_{{CpG}_{k}}*{M}_{{CpG}_{ki}}$. Each MRS was then included as a predictor in two separate Weibull AFT models—each adjusted for surrogate variables, age, race/ethnicity, and batch—to assess its association with time-to-progression.

#### Sensitivity analysis

We performed a sensitivity analysis to assess the analytic impact of collapsing multiple race/ethnicity groups into one “Other” designation. To accomplish this, we restricted our study sample to only those participants who identified as “non-Hispanic White” or “Black or African American” and re-performed the analytic steps for the “targeted analysis.”

All statistical analyses were conducted using R version 4.0.1 (Vienna, Austria).

## Results

### Participant characteristics

The distributions of socio-demographic and clinical characteristics for the 289 participants are displayed in Table 1.

**Table 1:** Characteristics of 289 colposcopy referral participants with ≤CIN1 enrollment histology in the CINCS study

		**Enrollment histology**^a^		
**Characteristic**	**Overall** **N (%)**^b^	**No CIN** **N (%)**	**CIN1** **N (%)**	***p-value***^c^
**Total**	289	186	103	
**Age (years)**				
*Median (Range)*	*29.2 (21.0–69.5)*	*31.4 (21.3–69.5)*	*27.5 (21.0–64.4)*	
21–24	67 (23.2%)	31 (16.7%)	36 (35.0%)	**< *0.01***
25–29	91 (31.5%)	58 (31.2%)	33 (32.0%)	
30 +	131 (45.3%)	97 (52.2%)	34 (33.0%)	
**High-risk HPV**				
Positive	185 (64.0%)	112 (60.2%)	73 (70.9%)	*0.07*
Negative	104 (36.0%)	74 (39.8%)	30 (29.1%)	
**Referral cytology**^d^				
LSIL	171 (59.2%)	98 (52.7%)	73 (72.3%)	***0.04***
ASCUS	78 (27.0%)	59 (31.7%)	19 (18.8%)	
ASC-H	21 (7.3%)	17 (9.1%)	4 (4.0%)	
LSIL-H	8 (2.8%)	5 (2.7%)	3 (3.0%)	
HSIL	4 (1.4%)	3 (1.6%)	1 (1.0%)	
Normal or Other	5 (1.7%)	4 (2.2%)	1 (1.0%)	
**Race/Ethnicity**^e^				
Non-Hispanic White	132 (45.7%)	85 (45.7%)	47 (45.6%)	*0.58*
Black	130 (45.0%)	86 (46.2%)	44 (42.7%)	
Other	27 (9.3%)	15 (8.1%)	12 (11.7%)	
**Current smoking**				
No	238 (82.4%)	147 (79.0%)	91 (88.3%)	*0.05*
Yes	51 (17.6%)	39 (21.0%)	12 (11.7%)	
**Current hormonal contraceptive use**^f^				
No	207 (71.6%)	143 (76.9%)	64 (62.1%)	***0.01***
Yes	82 (28.4%)	43 (23.1%)	39 (37.9%)	
**Parity**				
Nulliparous	142 (49.1%)	92 (50.0%)	50 (48.5%)	*0.56*
Primiparous (1)	63 (21.8%)	43 (23.4%)	20 (19.4%)	
Multiparous (2+)	82 (28.4%)	49 (26.6%)	33 (32.0%)	

^a^CIN = cervical intraepithelial neoplasia; CIN1 = low-grade CIN

^b^Numbers may not add up to total sample size due to missing data. Column percentages calculated as percent of non-missing for each characteristic. N missing: Referral cytology: *n* = 2; parity: *n* = 2

^c^Chi-square test *p*-value; in the case of cell size < 5, Fisher’s exact test was used

^d^LSIL = low-grade squamous intraepithelial lesion; ASCUS = atypical squamous cells of undetermined significance; ASC-H = atypical squamous cells, cannot exclude high-grade squamous intraepithelial lesion; LSIL-H = LSIL, cannot exclude high-grade squamous intraepithelial lesion; HSIL = high-grade squamous intraepithelial lesion; “Other” referral cytology includes unknown or inconclusive cytology results

^e^ Participants self-classified their race and ethnicity in a multiple-choice questionnaire at enrollment; “Other” includes self-classifications of “Asian/Pacific Islander” (*n* = 14) and “Hispanic White” (*n* = 13)

^f^ Hormonal contraceptives include oral, patch, injectable, and implant contraceptives

Median age at enrollment was 29.2 years, and 64.0% of participants were positive for hrHPV. Over half of participants identified as a race/ethnicity other than non-Hispanic White: 45.7% non-Hispanic White, 45.0% Black or African American, 4.8% Asian or Pacific Islander, and 4.5% Hispanic White. The most common Pap result was LSIL (59.2%), followed by ASCUS (27.0%), ASC-H (7.3%), LSIL-H (2.8%), and HSIL (1.4%). At enrollment, 186 (64.4%) participants had no CIN on histology and 103 (35.6%) had CIN1. Comparing CIN1 to no CIN, distributions of hrHPV positivity, race/ethnicity, current smoking, and parity were similar. However, participants with CIN1 were more likely to be younger, using some form of hormonal contraception, and had a different distribution of referral cytology findings compared to those with no CIN. Participants who did not return for any follow-up visits were more likely to have no CIN at enrollment than those retained in the study (Table S1).

Median study follow-up time was 24.4 person-months (range 3.7–62.3). The average number of follow-up study visits (after the enrollment visit) per person was 2.2 (range 1–7), and the average time in between study visits was 12.3 months. Over the course of follow-up, there were 15 events of progression to CIN2+ (5.2% overall; 3.8% of no CIN histology and 7.8% of CIN1) (Table 2).

**Table 2:** Outcomes of 289 CINCS participants followed up to 5 years, overall and stratified by enrollment histology

		**Enrollment histology**	
**Outcome**	**Overall** **N (%)**^a^	**No CIN** **N (%)**^a^	**CIN1** **N (%)**^a^
**Total**	289	186	103
**Progression to high-grade**^**b**^	**15 (5.2%)**	**7 (3.8%)**	**8 (7.8%)**
CIN2	7 (2.4%)	3 (1.6%)	4 (3.9%)
CIN2-3	5 (1.7%)	2 (1.1%)	3 (2.9%)
CIN3	3 (1.0%)	2 (1.1%)	1 (1.0%)
**No progression to high-grade**	**274 (94.8%)**	**179 (96.2%)**	**95 (92.2%)**
Normal	215 (74.4%)	146 (78.5%)	69 (67.0%)
CIN1	10 (3.5%)	4 (2.2%)	6 (5.8%)
ASCUS	27 (9.3%)	14 (7.5%)	13 (12.6%)
LSIL	22 (7.6%)	15 (8.1%)	7 (6.8%)

^a^Percentages are column percentages

^b^Participants counted as “progressed” on first instance of a progression event and were censored thereafter. Histology alone was used to categorize progression events, but cytology results of ASCUS or LSIL were acceptable for non-progressors

### Targeted analysis: Pre-selected CpGs and CIN2 + risk

CpG sites of nine pre-selected genes were ultimately included in the targeted analysis: *CADM1, CCNA1, CDH1, DAPK1, FHIT, MAL, PAX1, RARB,* and *RASSF1.* A total of 77 CpGs across these nine genes were identified with Illumina annotation and are listed by gene and cluster in Table S2, and genomic positions of these CpG sites are listed in Table S3.

Table 3 shows associations between CpG methylation levels and time-to-progression to CIN2+ and associated hazard ratios from the targeted AFT models. Increasing methylation of *CADM1* cg03505501 and *RARB* Cluster 1 (comprised of cg01697477 and cg27574595) were associated with increased CIN2+ risk at FDR < 0.05. Each one-unit increase in the continuous M value of *CADM1* cg03505501 was associated with a TTER of 0.28 (95% CI 0.12, 0.63), or a progression time that is 72% shorter (*p* < 0.01; FDR = 0.03); this corresponds to a HR 3.72 (95% CI 1.39, 9,98). Each one-unit increase in the continuous M value of *RARB* Cluster 1 was associated with a TTER of 0.46 (95% CI 0.29, 0.71), or a progression time that is 54% shorter (*p* < 0.01; FDR = 0.01). Increasing methylation levels at three other CpGs also trended with increasing CIN2+ risk, with unadjusted *p*-values < 0.05 but FDRs (adjusted for multiple comparisons) ≥ 0.05: The TTERs for *DAPK1* cg14286732, *PAX1* cg07213060, and *PAX1* Cluster 1 were 0.35 (95% CI 0.15, 0.80; *p* = 0.01; FDR = 0.07), 0.27 (95% CI 0.09, 0.78; *p* = 0.02; FDR = 0.07), and 0.30 (95% CI 0.10, 0.93; *p* = 0.04; FDR = 0.14), respectively. In other words, each one-unit increase in methylation M value at these sites was associated with a 65%, 73%, and 70% faster time-to-CIN2+, respectively. Conversely, increasing methylation at *CDH1* Cluster 1 (comprised of cg26508465 and cg10313337) showed an inverse trend, exhibiting a slower time-to-CIN2+, with a TTER of 5.74 (95% CI 1.64, 20.20; *p* = 0.01; FDR = 0.05).

**Table 3:** Targeted analysis: Associations between CpG site methylation for 9 genes^a^ and time-to-progression to CIN2+ over 5 years

			**CpG-specific** **AFT model**^b^** output**				**Converted proportional hazards output**	
**Gene**	**CpG site**	**Model β parameter**	**TTER**^c^	**TTER** **95% CI**	***p*-value**	**FDR**^d^	**HR**^e^	**HR 95% CI**
***CADM1***	cg03505501	**-1.28**	**0.28**	**0.12, 0.63**	**< 0.01**	**0.03**	**3.72**	**1.39, 9.98**
	cg08066991	-0.04	0.96	0.41, 2.26	0.92	0.96	1.22	0.32, 4.71
	cg14030346	-0.02	0.98	0.33, 2.92	0.97	0.97	1.53	0.28, 8.44
	Cluster 1	-0.41	0.66	0.19, 2.34	0.52	0.80	3.95	0.43, 35.86
***CCNA1***	cg12571423	-0.31	0.73	0.30, 1.82	0.50	0.80	1.28	0.29, 5.53
	cg23687560	0.25	1.29	0.40, 4.19	0.67	0.86	0.81	0.15, 4.37
	cg27142924	0.05	1.05	0.57, 1.92	0.88	0.96	0.45	0.18, 1.14
	Cluster 1	-0.67	0.51	0.23, 1.15	0.11	0.27	1.33	0.39, 4.58
***CDH1***	Cluster 1	**1.75**	**5.74**	**1.64, 20.10**	**0.01**	0.05	**0.07**	**0.01, 0.58**
	Cluster 2	0.21	1.23	0.51, 3.02	0.64	0.86	0.76	0.20, 2.82
***DAPK1***	cg14286732	**-1.05**	**0.35**	**0.15, 0.80**	**0.01**	0.07	3.16	0.92, 10.83
	Cluster 1	-0.54	0.59	0.24, 1.40	0.23	0.53	3.66	0.74, 18.07
***FHIT***	cg15135842	-0.06	0.94	0.56, 1.57	0.81	0.96	0.84	0.35, 2.03
	Cluster 1	-0.34	0.71	0.32, 1.58	0.40	0.76	1.50	0.47, 4.79
	Cluster 2	-0.48	0.62	0.19, 2.05	0.43	0.76	0.79	0.10, 5.90
***MAL***	cg19762657	-0.34	0.71	0.32, 1.59	0.41	0.76	2.06	0.64, 6.67
	cg21245652	0.49	1.63	0.30, 8.73	0.57	0.82	0.38	0.02, 8.46
***PAX1***	cg07213060	**-1.30**	**0.27**	**0.09, 0.78**	**0.02**	0.07	**12.62**	**1.35, 117.88**
	Cluster 1	**-1.20**	**0.30**	**0.10, 0.93**	**0.04**	0.14	6.45	0.78, 53.50
***RARB***	Cluster 1	**-0.79**	**0.46**	**0.29, 0.71**	**< 0.01**	**0.01**	0.90	0.21, 3.87
	Cluster 2	-0.91	0.40	0.14, 1.18	0.10	0.27	2.83	0.38, 21.17
***RASSF1***	Cluster 1	-0.10	0.91	0.31, 2.64	0.86	0.96	1.65	0.27, 9.99
	Cluster 2	1.16	3.19	0.98, 10.41	0.05	0.18	0.11	0.01, 0.97

^a^CpG = 5’-cytosine-phosphate-guanine-3’; Genes selected a priori from a meta-analysis (El Aliani et al.) showing that methylation levels at the promoters of these genes were significantly higher in cervical cancer cases and cancer precursors compared to controls. CpG sites for each gene were clustered if they had a correlation of > 0.5 with other CpG sites for that gene. Methylation level for each cluster is the median of all individual CpG M values in that cluster; CpGs included in each cluster can be found in Table S1 and genomic locations of CpGs are listed in Table S5

^b^AFT = accelerated failure time; Weibull-distributed adjusted AFT models were fit separately for each individual CpG site/cluster

^c^TTER = time-to-event ratio = exp(β). The TTER is interpreted as the ratio in times-to-progression per one-unit increase in methylation M value at the given CpG site/cluster. A TTER < 1 indicates shorter (quicker) time-to-progression

^d^FDR = False discovery rate

^e^HR = hazard ratio; HRs derived from Weibull AFT model parameters

**Fig. 2:**
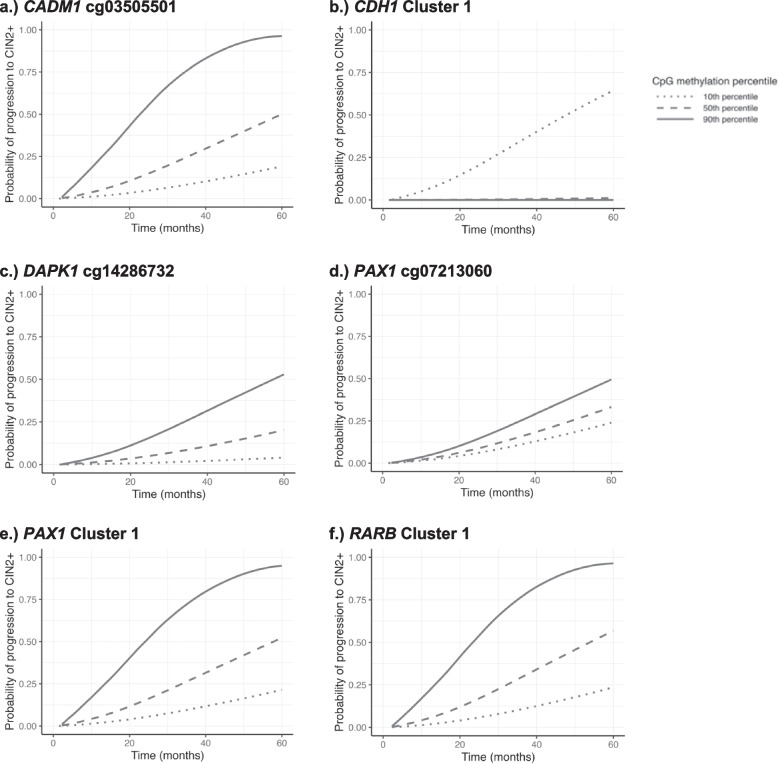
Targeted analysis: Risk curves for progression to CIN2+ for CpG sites with *p* < 0.05 Risk curves constructed with estimates from adjusted Weibull accelerated failure time models

For CpG sites with *p* < 0.05, risk curves showing the cumulative probability of progression to CIN2+ over time are displayed in Fig. 2. Curves for each site/cluster are plotted for three values of methylation M value: 10^th^, 50^th^, and 90^th^ percentiles, representing “lower”, median, and “higher” methylation levels, respectively. Figure 2 shows that higher methylation levels are associated with a higher probability (risk) of progression to CIN2+ for *CADM1* cg03505501, *DAPK1* cg14286732, *PAX1* cg07213060, *PAX1* Cluster 1, and *RARB* Cluster 1, and a lower probability (risk) of progression for *CDH1* Cluster 1.

### EWAS analysis: Epigenome-wide CpGs and CIN2+ risk

**Fig. 3:**
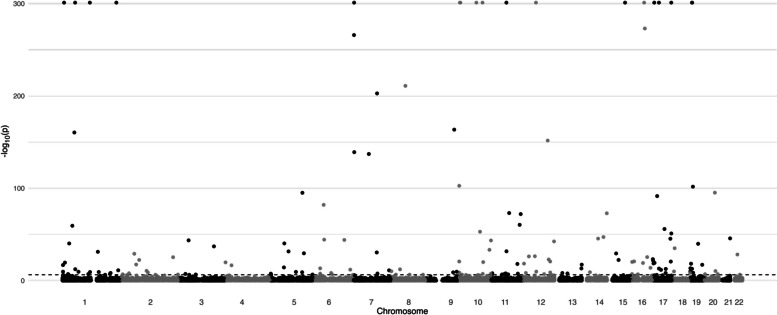
Exploratory analysis: Manhattan plot of EWAS results Unadjusted epigenome-wide *p*-values for association between continuous methylation levels at epigenome-wide CpGs and time-to-progression to CIN2+. EWAS *p*-values estimated from adjusted Weibull accelerated failure time models. Dashed horizontal line indicates the Bonferroni-adjusted epigenome-wide significance level

After all data processing steps and restricting to only those CpG sites shared among all three batches of arrays, a total of 101,078 CpGs were included in the epigenome-wide analysis. Figure 3 displays the Manhattan plot of the epigenome-wide analysis, where the -log_10_(p) of unadjusted *p*-values for all CpG sites are plotted by their chromosome number. There were 336 sites detected with FDR < 0.05. These sites, their genomic positions, associated genes, and gene functions are listed in Table S4. In the GSEA, no GO terms or KEGG pathways were enriched (no terms or pathways with FDR < 0.1 were detected).

### Methylation risk score

After randomly splitting the analytic sample, the training set included 154 participants (with 7 progression events), and the test set included 135 participants (with 8 progression events). Using an epigenome-wide approach, 6 CpG sites in the training set exhibited a Bonferroni *p* < 0.05: cg26118643, cg00688591, cg21584710, cg19474047, cg15883603, and cg04510564. In the test set, using the 6-CpG MRS as the main predictor in an adjusted AFT model, the MRS regression coefficient was -0.18, which corresponds to a TTER of 0.83 (*p* = 0.04) (Supplementary Table S5). Twenty-two CpG sites in the training set exhibited an FDR < 0.05. In the test set, the regression coefficient corresponding to this 22-CpG MRS was -0.04, which corresponds to a TTER of 0.96 (*p* = 0.96).

### Sensitivity analysis

There were 262 participants who identified as “non-Hispanic White” or “Black or African American” who were included in the sensitivity analysis. Targeted analysis results are displayed in Table S6 and are largely consistent with findings from the primary analysis.

## Discussion

This study is among the few to prospectively assess the risk of cervical disease progression associated with epigenome-wide methylation levels in screening-detected cervical abnormalities. This five-year prospective study investigated time-to-progression to CIN2+ associated with cervical sample methylation levels using time-to-event models in a higher-risk colposcopy referral population with ≤CIN1 at enrollment. We confirmed previously observed associations between *CADM1* and *RARB* CpG sites and time-to-progression to CIN2+, as well as trends supporting associations with *DAPK1 and PAX1* CpGs. Additionally, we identified 336 novel CpG sites in an exploratory epigenome-wide analysis of methylation levels and time-to-progression to CIN2+. These exploratory results may serve as the basis for future confirmatory studies or be included in meta-analyses to better elucidate their utility as clinical markers of cervical disease progression.

This study’s targeted analysis of several pre-selected genes assessed associations between methylation levels at these genes and cervical lesion severity or progression that have been previously observed in the literature. A meta-analysis conducted by El Aliani et al. found that methylation of promoter CpG sites increased as lesion severity increased from LSIL/CIN1 to HSIL/CIN2-3 to invasive cervical cancer for the genes we considered for this analysis: *CADM1, CCNA1, CDH1, CDKN2A, DAPK1, FHIT, MAL, PAX1, RARB*, and *RASSF1 *[15]. We utilized these findings and applied them in a time-to-event analysis to elucidate whether methylation levels found in low-grade lesions like LSIL and CIN1 are associated with time-to-progression to higher-grade lesions like CIN2-3. We found that increasing methylation level of *CADM1* cg03505501 and *RARB* Cluster 1 exhibited a positive association with progression. Other sites—*DAPK1* cg14286732, *PAX1* cg07213060, and *PAX1* Cluster 1*—*also trended toward similar associations with progression. Interestingly, we found that while most gene-related CpG sites exhibited positive associations with progression—where higher methylation levels conferred higher risks of progression—others showed opposite relationships. For example, a *CDH1*-associated CpG cluster had a TTER > 1, indicating that higher levels of methylation exhibited a protective association, with lower risks of progression over time. This supports that methylation at individual CpG sites associated with a particular gene may exert different biological effects on the gene’s functions [38, 39]. Thus, using a prospective study design with unique longitudinal time-to-event data, we replicated a subset of previously observed findings showing associations between CpG sites of specific cervical cancer-related genes and progression to CIN2+ among low-grade screening abnormalities.

Variations in the associations detected between CpG site methylation and cervical disease outcomes may be influenced by methodologies, geography, and demographics. For example, though we did not replicate other associations reported by El Aliani et al., this may be due to the use of different analytic methods, since we used time-to-event models, rather than a case–control study design, and continuous methylation levels as an exposure, rather than a dichotomized methylation status with a cut-off for “hypermethylation.” Second, our study population was a colposcopy referral population in the southeastern US, while the published meta-analysis included a variety of international studies, with a wide range of participant ascertainment methods. As methylation biomarkers continue to be investigated for their use in screening algorithms, it is important to study screening-detected lesions and follow people prospectively over time to best inform risk-based screening algorithms. Third, our cohort had relatively high percentages of non-Hispanic White and Black individuals and low percentages of individuals identifying as Asian or Hispanic/Latina; conversely, the meta-analysis was comprised of approximately 50% of studies conducted in Asian populations, 43% in populations of European descent, and 7% in African populations. Methylation levels and cervical disease epidemiology can vary greatly based on demographic composition of the study population—via largely social phenomena that manifest as biological differences in disease risk and health outcomes—which highlights the potential for variation in study findings and the importance of attempting to replicate studies like this in diverse populations.

Our exploratory epigenome-wide study identified 336 CpG sites whose methylation levels are associated with time-to-progression to CIN2+ that have not been previously identified in relation to cervical cancer. We searched EWAS Atlas for any studies that have previously implicated any of the top 100 sites by FDR detected in our EWAS [40, 41]. While none of the sites identified in this exploratory analysis have been previously associated with cervical cancer or its precursors, several have been previously associated with other cancer types, including oral squamous cell carcinoma [42] and hepatocellular carcinoma [43, 44]—which are both associated with infectious agents HPV and hepatitis, respectively—as well as thyroid cancer [45, 46], colorectal cancer and its adenomatous precursors [47], ovarian cancer [48], and prostate cancer [49]. These findings may be utilized to direct future studies of potential pathways and biomarkers for cervical disease progression. Replication of these findings in an external cohort will be needed. In the MRS analysis, we created two MRS versions: a 6-CpG MRS with CpG sites below a Bonferroni threshold and a 22-CpG MRS with CpG sites below an FDR threshold. While only the 6-CpG Bonferroni MRS attained a *p* < 0.05 in adjusted models, these findings highlight the promise of prospective methylation markers in this context.

Study strengths include the use of longitudinal data over five years to assess methylation-associated CIN2+ risk; this prospective study design is advantageous to quantify risk over time and is useful to inform clinical guidelines. Additionally, this study used Illumina methylation arrays, which quantify methylation levels at hundreds of thousands of CpG sites across the epigenome; this wide epigenomic coverage is important to continue to identify new CpG sites for further study. We also performed this array testing in low-grade lesions, or those with ≤CIN1 at baseline; since most screening-detected abnormalities fall into this “low-grade” category, this approach is especially useful for informing screening management guidelines. Finally, this study leveraged data from a unique US-based clinical cohort with most participants identifying as a non-white race or ethnicity—patient subpopulations that are historically underrepresented in many large genomic and epigenomic studies. Inclusion of BIPOC participants in clinical research is important to elucidate a representative understanding of the utility of cervical disease biomarkers and eventually creating appropriate clinical guidelines. Leveraging these unique strengths, our findings support the potential utility of both previously observed and novel methylation biomarkers to predict prospective risk of progression in low-grade cervical lesions to improve risk stratification in a multiracial US population.

A primary limitation of this study is the relatively small sample size, with a limited number of outcomes. This impacted the ability to make strong inferences due to large standard errors and wide 95% CIs and precluded us from stratifying the sample further by HPV type, for example. Second, methylation array testing was not performed on samples collected from follow-up visits. Thus, we were not able to assess whether methylation levels persisted over time in samples that eventually progressed. Third, selection bias due to attrition is a potential concern, since 157 participants potentially eligible for the analysis did not return to an affiliated clinic for a follow-up visit. Compared to those not retained in the study, those retained were more likely to have CIN1 at enrollment, which at least partially reflects guideline-concordant provider recommendations: Those with no CIN are at lower risk of progressing, and therefore are often recommended to return at longer intervals and are referred back to primary care providers, who may not have been in the Duke University medical system. Thus, our analysis may have captured those at a higher risk of progression. Fourth, this study recruited participants from a colposcopy referral population, which generally have higher baseline prevalence of cervical disease as compared to general screening populations, so results may not generalize to a general screening population. Fifth, since this was a clinical cohort, there was potential for outcome misclassification: biopsies were not always performed at each follow-up visit per conservative clinical practice, and there was no additional external expert pathology review of biopsy specimens. However, we restricted our outcome definition to only histologically-confirmed CIN2+ to more confidently capture true progressors. Sixth, although individuals identifying as Black comprised nearly half of our study sample, far fewer identified as Asian or Hispanic/Latina, and no one identified as Native American. This resulted in the need to collapse our race/ethnicity adjustment covariate to only two categories (“non-Hispanic White” and “race/ethnicity other than non-Hispanic White”); so, while it captured some variation in the data due to race/ethnicity, it likely did not capture all variation. Continuing to improve representation of BIPOC groups is an important priority for future screening studies assessing epigenomic biomarkers as predictors of risk. Finally, the analytic endpoint was defined as CIN2+, but CIN3 is more proximal to invasive cancer and thus would have strengthened our study. However, there is important clinical value in determining risk stratification at earlier timepoints—such as CIN2+—since clinical decision-making to undergo treatment or more frequent testing occurs at these earlier points. Indeed, CIN2 is often treated in clinical practice, thus precluding observation of many CIN3 cases.

In conclusion, the current study highlights the potential utility of methylation levels to predict progression to CIN2+ in cervical samples among patients with ≤CIN1. Methylation levels at specific CpG sites or for specific genes may be useful to identify patients who exhibit differential risks of progression to CIN2+ to further improve risk stratification for low-grade cervical lesions. Identifying methylation levels that confer higher or lower progression risk can help triage patients to more intensive or more conservative management, respectively, and thereby support the current “equal management for equal risk” US guidelines and improve the efficiency of cervical cancer screening cascades. For example, if several CpG sites are consistently found to be associated with disease progression, they can be included on a targeted panel that can be added to routine primary screening tests. Since our study was conducted in a colposcopy referral population, our findings would be most applicable to inform a future screening triage test, where methylation markers might further characterize the risk of progression of abnormal screening findings. This study in a diverse clinical cohort in the southeastern US contributes to the literature assessing risk attribution of CpG site methylation levels to progression to CIN2+ for cervical cancer screening. The novel sites identified here warrant further investigation in new cohorts, and further investigation of the applicability of these results to a general screening population is warranted.

### Supplementary Information

**Additional file 1: Figure S1.** Pre-processing pipeline for Illumina methylation data. **Table S1.** Comparison of enrollment characteristics of eligible participants by follow-up status. **Table S2.** CpG sites and CpG clusters constructed for selected genes. **Table S3.** Targeted Analysis: Genomic locations of CpG sites of 9 pre-selected genes included in targeted analysis. **Table S4.** Exploratory EWAS: CpG sites associated with time-to-progression to CIN2+ with epigenome-wide FDR <0.05 (*N*=336). **Table S5.** CpG sites included in methylation risk scores (MRS). **Table S6.** Sensitivity Analysis: Targeted associations between CpG site methylation for 9 genes and time-to-progression to CIN2+ over 5 years, restricted to 262 participants identifying as “non-Hispanic White” or “Black or African American” race.

## Data Availability

Data are not publicly available due to its sensitive nature (including protected health information of participants.) De-identified data may be made available upon reasonable request to the corresponding author for the purposes of replication of study results.
