# Supplementary material for: Epigenome-wide methylation and progression to high-grade cervical intraepithelial neoplasia (CIN2+): a prospective cohort study in the United States
Source: BMC Cancer. 2023 Nov 6;23:1072. doi: 10.1186/s12885-023-11518-6 (PMC10629205; doi:10.1186/s12885-023-11518-6)
Supplement: Supplementary file 1 — Additional file 1: Figure S1. Pre-processing pipeline for Illumina methylation data. Table S1. Comparison of enrollment characteristics of eligible participants by follow-up status. Table S2. CpG sites and CpG clusters constructed for selected genes. Table S3. Targeted Analysis: Genomic locations of CpG sites of 9 pre-selected genes included in targeted analysis. Table S4. Exploratory EWAS: CpG sites associated with time-to-progression to CIN2+ with epigenome-wide FDR <0.05 (N=336). Table S5. CpG sites included in methylation risk scores (MRS). Table S6. Sensitivity Analysis: Targeted associations between CpG site methylation for 9 genes and time-to-progression to CIN2+ over 5 years, restricted to 262 participants identifying as “non-Hispanic White” or “Black or African American” race. [file 12885_2023_11518_MOESM1_ESM.zip › CINCS2 Supplement_revised.docx]

**Figure S1. Pre-processing pipeline for Illumina methylation data**

DNA was extracted from 289 baseline cervical samples and was analyzed with the Illumina Human Methylation 450K (n=76) or EPIC 850K (n=213) BeadChip microarray. All microarray results were processed from raw .idat files. All pre-processing steps were performed separately on each of the three batches of methylation data (one 450K batch and two EPIC 850K batches) prior to statistical analyses.

**Methods Supplement: Illumina methylation data processing**

*Array data cleaning and quality control.* The following QC steps were performed on each of the three batches separately using the *ewastools* R package[1]: First, control metrics from the Illumina BeadArray Controls Reported Software Guide were checked for all samples; samples failing any of these metrics were excluded. Second, detection p-values were calculated, and undetected probes (detection p>0.01) were set to missing; samples where >10% of their CpG probes were undetected were excluded and probes where >10% of the samples were undetected were removed. A dye-bias correction was then applied. Next, the log-odds of being an outlier across all SNP probes was estimated, and all samples identified as SNP outliers were excluded. A total of 22 samples in the 450K batch and one sample in the second 850K batch were excluded due to failing the above QC metrics (no samples failed from the first 850K batch). A check for duplicate-genotype samples was performed and duplicates were removed, if found. Finally, additional CpG filters were applied to remove common polymorphic sites, polymorphic sites commonly identified in African American individuals, and probes with mapping issues, cross-reactivity, color-channel switching, and probes corresponding to SNPs and sex chromosomes.

*Additional array data processing.* After initial cleaning and QC, the following methylation data processing steps were applied: First, missing beta values were imputed using the k-Nearest Neighbors (KNN) method with the *ChAMP* R package.[2, 3] Second, beta values underwent beta-mixture quantile (BMIQ) normalization, also with *ChAMP*. At this point, beta values were converted to M values, which were used in subsequent analyses due to their improved statistical properties; no beta values were 0 or 1, so there were no issues with the conversion to M values. Then, the dataset of M values was adjusted for batch effects (plate, row, and column) using the *sva* package.[4] Finally, technical replicates—samples that underwent repeated methylation testing across the three batches—were used to remove poorly replicating probes. There were five samples replicated between Batch 1 and Batch 3 and four samples replicated between Batch 2 and Batch 3. The *CpGFilter* package in R was used to calculate intra-class correlation coefficients (ICC) between the probes for each replicated pairs of samples, where the ICC is takes on values between 0 and 1.[5] Probes with an ICC of <0.5 between the replicated samples were excluded. Replicate samples were then retained in the batch with the smaller sample size and removed from the batch with the larger sample size. Only CpG sites represented in all three batches were included in further analyses (i.e., the methylation data was reduced to those included on both the 450K and EPIC 850K platforms). Throughout data processing, we chose to adhere to more stringent cut-offs that resulted in higher numbers of probes being excluded, but allowed us to obtain more conservative estimates of true methylation signals. After data processing, a total of 101,078 CpGs were included in the epigenome-wide analyses below; though this represents a large reduction from the total number of sites originally included in the methylation arrays, it is congruent with final numbers of CpG sites remaining in analytic datasets after following similar processing procedures.[6]

**References:**

1. Heiss JA. EWAS Tools (ewastools) R Package Details. GitHub documentation. 2022. https://rdrr.io/github/hhhh5/ewastools/. Accessed 11 Dec 2022.

2. Tian Y, Morris T, Stirling L, Feber A, Teschendorff A, Chakravarthy A. ChAMP Package - R documentation: Chip Analysis Methylation Pipeline for Illumina HumanMethylation450 and EPIC. 2020.

3. Tian Y, Morris TJ, Webster AP, Yang Z, Beck S, Feber A, et al. ChAMP: updated methylation analysis pipeline for Illumina BeadChips. Bioinformatics. 2017;33:3982–4.

4. Leek JT, Johnson WE, Parker HS, Jaffe AE, Storey JD. The sva package for removing batch effects and other unwanted variation in high-throughput experiments. Bioinformatics. 2012;28:882.

5. Chen J. CpGFilter: CpG Filtering Method Based on Intra-Class Correlation Coefficients. CRAN R Documentation. 2017. https://cran.r-project.org/web/packages/CpGFilter/index.html. Accessed 11 Dec 2022.

6. Vanderlinden LA, Johnson RK, Carry PM, Dong F, DeMeo DL, Yang I V., et al. An effective processing pipeline for harmonizing DNA methylation data from Illumina’s 450K and EPIC platforms for epidemiological studies. BMC Res Notes. 2021;14:1–7.

**Table S1. Comparison of enrollment characteristics of eligible participants by follow-up status**

| **Enrollment characteristic** | **≥1 follow-up visit**  **N (%)**^a^ | **No follow-up visits**  **N (%)**^a^ | ***p*-value**^b^ |
| --- | --- | --- | --- |
| **Total** | 289 | 157 |  |
| **Enrollment histology** |  |  |  |
| No CIN | 186 (64.4%) | 118 (75.2%) | ***0.02*** |
| CIN1 | 103 (35.6%) | 39 (24.8%) |  |
| **Age (years)** |  |  |  |
| *Median (Range)* | *29.2 (21.0-69.5)* | *27.8 (21.1-67.6)* |  |
| 21-24 | 67 (23.2%) | 53 (33.8%) | *0.05* |
| 25-29 | 91 (31.5%) | 39 (24.8%) |  |
| 30+ | 131 (45.3%) | 65 (41.4%) |  |
| **High-risk HPV** |  |  |  |
| Positive | 185 (64.0%) | 96 (61.1%) | *0.55* |
| Negative | 104 (36.0%) | 61 (38.9%) |  |
| **Referral cytology** |  |  |  |
| LSIL | 171 (59.2%) | 82 (52.2%) | *0.64* |
| ASCUS | 78 (27.0%) | 55 (35.0%) |  |
| ASC-H | 21 (7.3%) | 10 (6.4%) |  |
| LSIL-H | 8 (2.8%) | 5 (3.2%) |  |
| HSIL | 4 (1.4%) | 2 (1.3%) |  |
| Normal or Other^c^ | 5 (1.7%) | 3 (1.9%) |  |
| **Race/Ethnicity** |  |  |  |
| Non-Hispanic White | 132 (45.7%) | 70 (44.9%) | *0.95* |
| Black or African American | 130 (45.0%) | 70 (44.9%) |  |
| Other^d^ | 27 (9.3%) | 16 (10.3%) |  |
| **Currently smoking** |  |  |  |
| No | 238 (82.4%) | 128 (83.7%) | *0.73* |
| Yes | 51 (17.6%) | 25 (16.3%) |  |
| **Current hormonal contraceptive use**^e^ |  |  |  |
| No | 207 (71.6%) | 113 (72.0%) | *0.94* |
| Yes | 82 (28.4%) | 44 (28.0%) |  |
| **Parity** |  |  |  |
| Nulliparous | 142 (49.1%) | 79 (52.0%) | *0.88* |
| Primiparous (1) | 63 (21.8%) | 31 (20.4%) |  |
| Multiparous (2+) | 82 (28.4%) | 42 (27.6%) |  |
| ^a^ Numbers may not add up to total sample size due to missing data. Percentages calculated as percent of non-missing.  ^b^ Chi-square test p-value, comparing those retained to those not retained; in the case of cell size <5, Fisher’s exact test used  ^c^ Other referral cytology includes unknown or inconclusive results.  ^d^ Participants self-classified race and ethnicity in multiple-choice question at enrollment; “Other” includes “Asian/Pacific Islander” and “Hispanic White”.  ^e^ Hormonal contraceptives include oral, patch, injectable, and implant contraceptives. | | | |

**Table S2. CpG sites and CpG clusters constructed for selected genes**

| **Gene**^a^ | **Cluster**^b^ | **CpG sites**^c^ | **Gene biological role** | **Meta-analysis observations** |
| --- | --- | --- | --- | --- |
| ***CADM1*** | Cluster 1 | cg01948062, cg24749470, cg11594811 | Cell adhesion | Hypermethylation in cervical cancer cases and precursors + increasing hypermethylation in cervical lesions of increasing severity |
|  | -- | cg08066991 |  |  |
|  | -- | cg03505501 |  |  |
|  | -- | cg14030346 |  |  |
| ***CCNA1*** | Cluster 1 | cg13711394, cg21754388, cg23831735, cg05120331, cg14089714, cg07962128, cg21587066, cg24154053 | Cell cycle and apoptosis mediator |  |
|  | -- | cg27142924 |  |  |
|  | -- | cg23687560 |  |  |
|  | -- | cg12571423 |  |  |
| ***CDH1*** | Cluster 1 | cg26508465, cg10313337 | Cell adhesion |  |
|  | Cluster 2 | cg11667754, cg17655614, cg01251360, cg07762788, cg09406989, cg24765079, cg08051386 |  |  |
| ***CDKN2A*** | -- | -- | Cell cycle control and regulation |  |
| ***DAPK1*** | Cluster 1 | cg14014720, cg13765778 | Cell cycle control and regulation |  |
|  | -- | cg14286732 |  |  |
| ***FHIT*** | Cluster 1 | cg08195983, cg21541638 | Cell cycle control and regulation |  |
|  | Cluster 2 | cg22215728, cg16806041, cg01556706, cg20366397, cg17894779, cg22533480, cg10131075, cg08223225, cg11815980, cg03060986, cg10763247 |  |  |
|  | -- | cg15135842 |  |  |
| ***MAL*** | -- | cg21245652 | Protein transport |  |
|  | -- | cg19762657 |  |  |
| ***PAX1*** | Cluster 1 | cg22763649, cg01446894, cg02155658, cg26463200, cg04543233, cg06654901, cg10629004, cg16309876 | Cell differentiation |  |
|  | -- | cg07213060 |  |  |
| ***RARB*** | Cluster 1 | cg01697477, cg27574595 | Cell signaling during cell growth and differentiation | Hypermethylation in cervical cancer cases and precursors only |
|  | Cluster 2 | cg21646032, cg11151405, cg21902772, cg00371702 |  |  |
| ***RASSF1*** | Cluster 1 | cg02930432, cg06172942, cg21908110, cg24859722, cg06821120, cg21418575, cg06980053, cg07130266, cg01932734, cg22090713, cg19854901, cg20119308, cg05546296, cg05989693 | Cell signaling | Hypermethylation in cervical cancer cases and precursors + increasing hypermethylation in cervical lesions of increasing severity |
|  | Cluster 2 | cg00980904, cg19811994, cg06174454 |  |  |

^a^ Genes selected *a priori* from a meta-analysis (El Aliani et al.) showing that methylation levels at the promoters of these genes were significantly higher in cervical cancer cases and cancer precursors compared to controls. El Aliani et al. found 10 genes with these findings, but there were no CpG sites for *CDKN2A* included in our analysis, so our analysis includes only nine genes.

^b^ CpG sites for each gene were clustered if they had a correlation of >0.5 with other CpG sites for that gene. Methylation level for each cluster is the median of all individual CpG M values in that cluster.

^c^ CpG site locations (chromosome number and position) are listed in Supplementary Table S3

**Table S3. Targeted Analysis: Genomic locations of CpG sites of 9 pre-selected genes included in targeted analysis** *(excel file)*

**Table S4. Exploratory EWAS: CpG sites associated with time-to-progression to CIN2+ with epigenome-wide FDR <0.05 (N=336)** *(excel file)*

**Table S5. CpG sites included in methylation risk scores (MRS)**^a^

| **Identification criterion** | **CpG sites** | **Gene** | **Chromosome** | **Position** | **Model coefficient** | **p-value** |
| --- | --- | --- | --- | --- | --- | --- |
| ***Bonferroni p<0.05*** | cg26118643 |  | 1 | 8009079 | -0.18  (TTER=0.83) | ***0.04*** |
|  | cg00688591 |  | 13 | 113097140 |  |  |
|  | cg21584710 | *DNAI2* | 17 | 72306141 |  |  |
|  | cg19474047 | *ANGPTL6* | 19 | 10207448 |  |  |
|  | cg15883603 | *SORBS2* | 4 | 186622408 |  |  |
|  | cg04510564 |  | 7 | 27034796 |  |  |
| ***FDR <0.05*** | cg26118643 |  | 1 | 8009079 | -0.04  (TTER=0.96) | *0.96* |
|  | cg06094482 |  | 1 | 40158708 |  |  |
|  | cg09023743 |  | 1 | 95045763 |  |  |
|  | cg07682515 |  | 10 | 134838014 |  |  |
|  | cg02895447 | *SYT7* | 11 | 61335600 |  |  |
|  | cg20964304 | *PKNOX2* | 11 | 125040416 |  |  |
|  | cg23105471 | *CTDSP2* | 12 | 58241215 |  |  |
|  | cg00688591 |  | 13 | 113097140 |  |  |
|  | cg26344747 | *MAP2K3* | 17 | 21193332 |  |  |
|  | cg20935841 | *MED24; SNORD124* | 17 | 38183170 |  |  |
|  | cg21584710 | *DNAI2* | 17 | 72306141 |  |  |
|  | cg19474047 | *ANGPTL6* | 19 | 10207448 |  |  |
|  | cg00174992 | *NOSTRIN* | 2 | 169659121 |  |  |
|  | cg24640676 | *C21orf29; KRTAP10-9* | 21 | 46046183 |  |  |
|  | cg12074966 | *GPR156* | 3 | 119889512 |  |  |
|  | cg13348530 | *SORCS2* | 4 | 7283184 |  |  |
|  | cg20774191 |  | 4 | 165851859 |  |  |
|  | cg15883603 | *SORBS2* | 4 | 186622408 |  |  |
|  | cg05411199 | *C6orf145* | 6 | 3749715 |  |  |
|  | cg25980484 | *KIAA1949* | 6 | 30653512 |  |  |
|  | cg04510564 |  | 7 | 27034796 |  |  |
|  | cg25483003 | *ENTPD2* | 9 | 139948856 |  |  |

^a^ CpG sites identified in training set (N=154) EWAS with Bonferroni p<0.05 or FDR <0.05. CpG sites meeting these criteria were used in the test set (N=135) to create each respective MRS. Each MRS was included as a predictor in separate adjusted models, and corresponding model coefficients and p-values are displayed.

**Table S6. Sensitivity Analysis: Targeted associations between CpG site methylation for 9 genes**^a^ **and time-to-progression to CIN2+ over 5 years, restricted to 262 participants identifying as “non-Hispanic White” or “Black or African American” race**

|  |  |  |  |  | | **CpG-specific**  **AFT model**^b^ **output** | | | | | | | |  | **Converted proportional hazards output** | | |
| --- | --- | --- | --- | --- | --- | --- | --- | --- | --- | --- | --- | --- | --- | --- | --- | --- | --- |
| **Gene** | **CpG site** |  | **Model β parameter** | |  | | **TTER**^c^ |  | **TTER**  **95% CI** |  | **p-value** |  | **FDR**^d^ |  | **HR**^e^ |  | **95% CI** |
| ***CADM1*** | cg03505501 |  | **-1.33** | |  | | **0.27** |  | **0.11, 0.62** |  | **<0.01** |  | **0.02** |  | **4.39** |  | **1.43, 13.45** |
|  | cg08066991 |  | 0.24 | |  | | 1.27 |  | 0.49, 3.32 |  | 0.62 |  | 0.94 |  | 0.61 |  | 0.13, 2.85 |
|  | cg14030346 |  | 0.09 | |  | | 1.09 |  | 0.31, 3.87 |  | 0.89 |  | 0.95 |  | 0.91 |  | 0.12, 7.10 |
|  | Cluster 1 |  | -0.20 | |  | | 0.82 |  | 0.21, 3.14 |  | 0.77 |  | 0.95 |  | 3.61 |  | 0.33, 39.99 |
| ***CCNA1*** | cg12571423 |  | -0.37 | |  | | 0.69 |  | 0.256, 1.86 |  | 0.46 |  | 0.82 |  | 1.04 |  | 0.23, 4.62 |
|  | cg23687560 |  | 0.85 | |  | | 2.34 |  | 0.53, 10.32 |  | 0.26 |  | 0.54 |  | 0.35 |  | 0.05, 2.36 |
|  | cg27142924 |  | 0.03 | |  | | 1.03 |  | 0.53, 2.02 |  | 0.92 |  | 0.95 |  | 0.42 |  | 0.16, 1.13 |
|  | Cluster 1 |  | -0.69 | |  | | 0.50 |  | 0.18, 1.36 |  | 0.17 |  | 0.40 |  | 1.47 |  | 0.34, 6.32 |
| ***CDH1*** | Cluster 1 |  | **1.77** | |  | | **5.86** |  | **1.56, 22.09** |  | **0.01** |  | 0.07 |  | **0.07** |  | **0.01, 0.84** |
|  | Cluster 2 |  | -1.28 | |  | | 0.28 |  | 0.05, 1.51 |  | 0.14 |  | 0.35 |  | 7.90 |  | 0.83, 75.11 |
| ***DAPK1*** | cg14286732 |  | -0.84 | |  | | 0.43 |  | 0.17, 1.12 |  | 0.08 |  | 0.28 |  | 2.42 |  | 0.58, 10.13 |
|  | Cluster 1 |  | -0.30 | |  | | 0.74 |  | 0.30, 1.85 |  | 0.52 |  | 0.85 |  | 1.58 |  | 0.30, 8.26 |
| ***FHIT*** | cg15135842 |  | 0.14 | |  | | 1.15 |  | 0.63, 2.12 |  | 0.65 |  | 0.94 |  | 0.46 |  | 0.16, 1.32 |
|  | Cluster 1 |  | -0.10 | |  | | 0.90 |  | 0.38, 2.16 |  | 0.82 |  | 0.95 |  | 0.71 |  | 0.18, 2.83 |
|  | Cluster 2 |  | -0.22 | |  | | 0.80 |  | 0.21, 3.03 |  | 0.74 |  | 0.95 |  | 0.63 |  | 0.06, 6.95 |
| ***MAL*** | cg19762657 |  | -0.43 | |  | | 0.65 |  | 0.27, 1.57 |  | 0.34 |  | 0.65 |  | 2.51 |  | 0.73, 8.57 |
|  | cg21245652 |  | -0.10 | |  | | 0.91 |  | 0.12, 6.70 |  | 0.93 |  | 0.95 |  | 0.30 |  | 0.01, 10.25 |
| ***PAX1*** | cg07213060 |  | **-1.28** | |  | | **0.28** |  | **0.09, 0.89** |  | **0.03** |  | 0.18 |  | 9.33 |  | 0.93, 93.12 |
|  | Cluster 1 |  | **-1.34** | |  | | **0.26** |  | **0.07, 0.94** |  | **0.04** |  | 0.18 |  | 4.93 |  | 0.45, 53.78 |
| ***RARB*** | Cluster 1 |  | **-0.86** | |  | | **0.42** |  | **0.27, 0.66** |  | **<0.01** |  | **<0.01** |  | 0.43 |  | 0.12, 1.56 |
|  | Cluster 2 |  | -1.03 | |  | | 0.36 |  | 0.11, 1.13 |  | 0.08 |  | 0.28 |  | 2.62 |  | 0.28, 24.52 |
| ***RASSF1*** | Cluster 1 |  | 0.04 | |  | | 1.04 |  | 0.31, 3.47 |  | 0.95 |  | 0.95 |  | 0.85 |  | 0.12, 5.99 |
|  | Cluster 2 |  | 0.95 | |  | | 2.58 |  | 0.76, 8.74 |  | 0.13 |  | 0.35 |  | 0.25 |  | 0.03, 2.46 |

^a^ CpG = 5’-cytosine-phosphate-guanine-3’; Genes selected *a priori* from a meta-analysis (El Aliani et al.) showing that methylation levels at the promoters of these genes were significantly higher in cervical cancer cases and cancer precursors compared to controls. CpG sites for each gene were clustered if they had a correlation of >0.5 with other CpG sites for that gene. Methylation level for each cluster is the median of all individual CpG M values in that cluster; CpGs included in each cluster can be found in Table S1 and genomic locations of CpGs are listed in Table S5.

^b^ AFT = accelerated failure time; Weibull-distributed adjusted AFT models were fit separately for each individual CpG site/cluster

^c^ TTER = time-to-event ratio = exp(β). The TTER is interpreted as the ratio in times-to-progression per one-unit increase in methylation M value at the given CpG site/cluster. A TTER<1 indicates shorter (quicker) time-to-progression.

^d^ FDR = False discovery rate

^e^ HR = hazard ratio; HRs derived from Weibull AFT model parameters.
